# Structural Understanding of Interleukin 6 Family Cytokine Signaling and Targeted Therapies: Focus on Interleukin 11

**DOI:** 10.3389/fimmu.2020.01424

**Published:** 2020-07-16

**Authors:** Riley D. Metcalfe, Tracy L. Putoczki, Michael D. W. Griffin

**Affiliations:** ^1^Department of Biochemistry and Molecular Biology, Bio21 Molecular Science and Technology Institute, The University of Melbourne, Parkville, VIC, Australia; ^2^Personalised Oncology Division, The Walter and Eliza Hall Institute of Medical Research, Parkville, VIC, Australia; ^3^Department of Medical Biology, The University of Melbourne, Parkville, VIC, Australia

**Keywords:** cytokine, interleukin, IL-11, IL-6, JAK, STAT, structural biology, drug development

## Abstract

Cytokines are small signaling proteins that have central roles in inflammation and cell survival. In the half-century since the discovery of the first cytokines, the interferons, over fifty cytokines have been identified. Amongst these is interleukin (IL)-6, the first and prototypical member of the IL-6 family of cytokines, nearly all of which utilize the common signaling receptor, gp130. In the last decade, there have been numerous advances in our understanding of the structural mechanisms of IL-6 family signaling, particularly for IL-6 itself. However, our understanding of the detailed structural mechanisms underlying signaling by most IL-6 family members remains limited. With the emergence of new roles for IL-6 family cytokines in disease and, in particular, roles of IL-11 in cardiovascular disease, lung disease, and cancer, there is an emerging need to develop therapeutics that can progress to clinical use. Here we outline our current knowledge of the structural mechanism of signaling by the IL-6 family of cytokines. We discuss how this knowledge allows us to understand the mechanism of action of currently available inhibitors targeting IL-6 family cytokine signaling, and most importantly how it allows for improved opportunities to pharmacologically disrupt cytokine signaling. We focus specifically on the need to develop and understand inhibitors that disrupt IL-11 signaling.

## Introduction

### Cytokine Signaling—A Brief History

In 1957, interferons were the first cytokines to be identified as secreted protein products induced following virus infection (1). In the subsequent decades, similar proteins, including the colony stimulating factors (CSFs) (2–4), Interleukin (IL)-2 (5, 6), and IL-3 (7, 8) were identified as secreted molecules able to support the growth of various hematopoietic cell linages *in vitro*. In 1974, the broad term “cytokine” was introduced (9) and in 1979 the term “interleukin” was introduced to standardize the names of the proteins now known as IL-1 and IL-2 (10). Over the next decade, radiolabelling studies revealed that cytokines bound distinct and unique receptors on the cell surface (11). It was also revealed that some cytokines, such as granulocyte-macrophage CSF (GM-CSF), IL-5 and IL-3 compete for a low-affinity receptor (12, 13), foreshadowing the identification of the β common receptor.

Following the discovery of the first cytokines, the mechanisms of intracellular signal transduction by cytokines remained elusive. The first transcriptional activator to be well-characterized was interferon-stimulated gene factor 3 (ISGF3), a multi-component protein complex consisting of what is now known as signal transducer and activator of transcription (STAT)1 and STAT2 (14, 15). Subsequently, related STAT proteins were identified as being activated *via* cytokine stimulation (16, 17). It was also shown that these factors were tyrosine phosphorylated (18, 19) on cytokine activation. The kinases responsible for this phosphorylation, the Janus kinases (JAKs) were first identified through a PCR screen of a murine hematopoietic cell line (20, 21). Their significance was unclear until the early 1990s, when they were shown to be activated as a result of cytokine binding and to phosphorylate the transcription factors that were already identified as key for interferon signal transduction (22). Subsequently, different members of the JAK family were found to be responsible for signal transduction by numerous cytokines (23–25). In 1997, the negative feedback regulators of the pathway, the suppressors of cytokine signaling (SOCS) proteins were identified (26–28). The key components of cytokine signaling using the JAK-STAT pathway were thus understood by the late 1990s, although many of the detailed molecular mechanisms are still unknown and remain under intense investigation today.

IL-6 family cytokines belong to a large group that signal *via* the JAK-STAT pathway, are characterized by a four α-helical bundle structure, and share receptors with similar structures consisting of several fibronectin type III (Fn3) and immunoglobulin-like (Ig-like) domains (29–31). Other cytokines, such as the IL-1/IL-18 family and the TNF-α family are structurally distinct from the four-α helical bundle family (32), utilize different signaling mechanisms, and are thus beyond the scope of this review. Conversely, several protein hormones, such as leptin, growth hormone (GH), prolactin and erythropoietin (EPO) utilize similar signal transduction mechanisms, are structurally related to the four-α helical bundle cytokines, and are thus best categorized alongside them (30, 33). The discovery of GH and EPO predate that of the interferons by several decades (34–37), but they were not recognized as related until they were cloned, sequenced, and significant sequence homology was noted between the receptors, GHR and EPOR (38, 39).

### The Structure of Cytokines and Their Receptors

**Figure 1:**
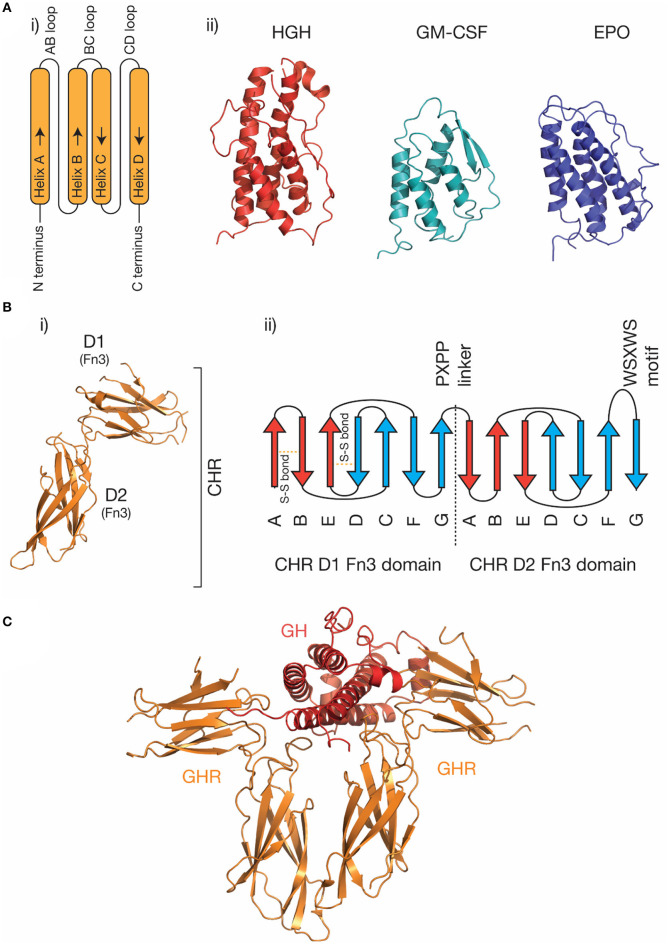
The structure of cytokines and receptors. **(A)** (i) A schematic of the four-α helical bundle topology of hematopoietic cytokines, (ii) cartoon representations of the structures of several representative cytokines; human growth hormone [PDB ID: 1HGU (40)], GM-CSF [PDB ID: 1CSG (41)], and erythropoietin [PDB ID: 1BUY (42)]. **(B)** The structure of the growth hormone receptor [PDB ID: 2AEW (43)]. The two Fn3 domains that make up the CHR are indicated, and a typical topology (30) for the two Fn3 domains in the CHR is shown in (ii). The conserved disulfide bonds in the N-terminal domain, the linker sequence, and the conserved WSXWS motif are indicated. **(C)** The structure of the growth hormone/growth hormone receptor complex [PDB ID: 3HHR (44)].

The four-α helical bundle cytokine family is the largest cytokine family. Both class I cytokines (e.g., GH, IL-6, IL-11) and class II cytokines (e.g., IFN-α, IL-10) utilize receptors that are broadly similar in structure and initiate similar intracellular signaling mechanisms (29). Cytokines from both classes are characterized by a compact α-helical bundle formed by four anti-parallel α-helices, arranged in an up-up-down-down topology (29, 31). This arrangement of helices necessitates long loops joining the helices (Figure 1A). Secondary structure in the loops is common, for example, the loop joining the C and D helices in IL-6 (the CD loop) contains a short α-helix (45), and in IL-4 (46) and GM-CSF (41), the AB and CD loops form a small anti-parallel β-sheet on the same face of the cytokine (Figure 1A). The topology of the four-α helical bundle fold provides a large surface area for the cytokine to bind its receptors.

Cytokine receptors are generally modular, single-pass transmembrane proteins, with a large extracellular region consisting of multiple all-β Ig-like domains and Fn3 domains (33). Both domains possess a β-sandwich structure, with two anti-parallel β sheets (Figure 1B). The exception are the IL-2Rα/IL-15Rα receptors, which consist of two all-β sushi domains, unrelated to the Ig and Fn3 domains comprising other cytokine receptors (33, 47, 48). The cytokine binding domains of the receptors consist of two Fn3 domains at approximately a 90° angle, forming the cytokine binding homology region (CHR) (30). Cytokines bind at the junction of these two domains. Each of the two domains of the CHR possess conserved features, the N-terminal domain of the CHR has two conserved disulphide bonds, and in class I cytokine receptors of the C-terminal domain of the CHR has a highly conserved Trp-Ser-X-Trp-Ser motif (WSXWS) motif (30). The WSXWS motif generally forms a “ladder” consisting of cation-π interactions between the tryptophan and arginine side chains. The precise structural role of the WSXWS motif is still unclear. It may stabilize the receptor, since mutations in the WSXWS motif result in a non-functional receptor (49, 50), and a rare genetic disease results from a mutation in the WSXWS motif of GHR (51). In IL-21Rα, the first Trp of the WSXWS motif is C-mannosylated and this modified Trp forms stabilizing interactions with other glycans and amino acid residues in the structure (52). The extensive glycosylation, both Trp C-mannosylation, and N-linked glycosylation gives IL-21Rα the structure of an “A-frame,” with a glycan chain forming a bridge between the two domains in the receptor. Similar Trp C-mannosylation has been detected in the p40 subunit of IL-12 by mass spectrometry (53), but has not been observed in crystal structures which include p40 (54–56), possibly reflecting incomplete incorporation of the modification in recombinant protein. Recent studies have suggested that, in addition to being a stabilizing structural element, the WSXWS motif undergoes a conformational change on cytokine binding, suggesting it has a role in receptor activation (57).

Beyond the CHR, many cytokine receptors have additional extracellular domains. These domains have varied roles, for example in correctly orienting the receptor to allow the activation of intracellular kinases (58), to facilitate cytokine binding (59), or to modulate intracellular trafficking to the membrane (60). While, most cytokine receptors are single-pass transmembrane proteins, an exception is the ciliary neurotrophic factor (CNTF) receptor, which is lipid anchored (61). The structures of cytokine receptor transmembrane domains have been solved, generally by nuclear magnetic resonance (NMR) spectroscopy (62–64). Single-pass transmembrane cytokine receptors also possess an intracellular domain that is assumed to be highly dynamic (65, 66). In the case of signal-transducing cytokine receptors, the intracellular domain binds signal transducing molecules, such as the JAKs, STATs, and the SOCS proteins.

Understanding the molecular details of cytokine engagement requires detailed structural knowledge of the complexes formed by cytokines and receptors. The first cytokine/receptor complex structure solved was the GH:GHR complex in 1992 (Figure 1C), which revealed GH bound to a dimer of GHR (44). The most surprising feature of the structure was the observation that two chemically distinct binding sites on GH bind similar epitopes on GHR. Following the GH:GHR structure, more complex structures followed, such as the tetrameric viral IL-6 (67) complex, the hexameric IL-6 (68) complex, and the dodecameric GM-CSF (69) complex, providing a more thorough understanding of cytokine/receptor engagement from several cytokine families. To date, no high-resolution structures have been solved that include the transmembrane or intracellular regions of cytokine receptors, although low-resolution negative-stain electron microscopy studies have captured the overall organization of these complexes (65, 70, 71).

The use of shared signal transducing receptors by cytokines is common. For example, three cytokines utilize the common β chain (β_c_), IL-3, IL-5, and GM-CSF (72), six cytokines utilize the common γ chain (γ_c_), IL-2, IL-7, IL-9, IL-13, IL-15, and IL-21 (73), and more than ten cytokines utilize glycoprotein (gp)130, including IL-6, IL-11, leukemia inhibitory factor (LIF), CNTF and oncostatin M (OSM) (74, 75). As structures have now been solved of several representative cytokines from these families, the mechanisms of shared receptor use have begun to be understood. For example, the γ_c_ receptor has a large binding surface in the CHR, allowing it to bind structurally diverse cytokines (48, 73), in contrast, gp130 has a structurally rigid, chemically diverse binding surface at the CHR, with different gp130-binding cytokines interacting with different but overlapping regions of the surface (76). In shared receptor systems, cytokine-specific receptors with restricted expression, such as IL-6Rα or IL-15Rα, serve to limit the activity of cytokines to specific target cells despite their utilization of similar intracellular signaling pathways.

### Intracellular Signal Transduction by Cytokines—The JAK-STAT Pathway

**Figure 2:**
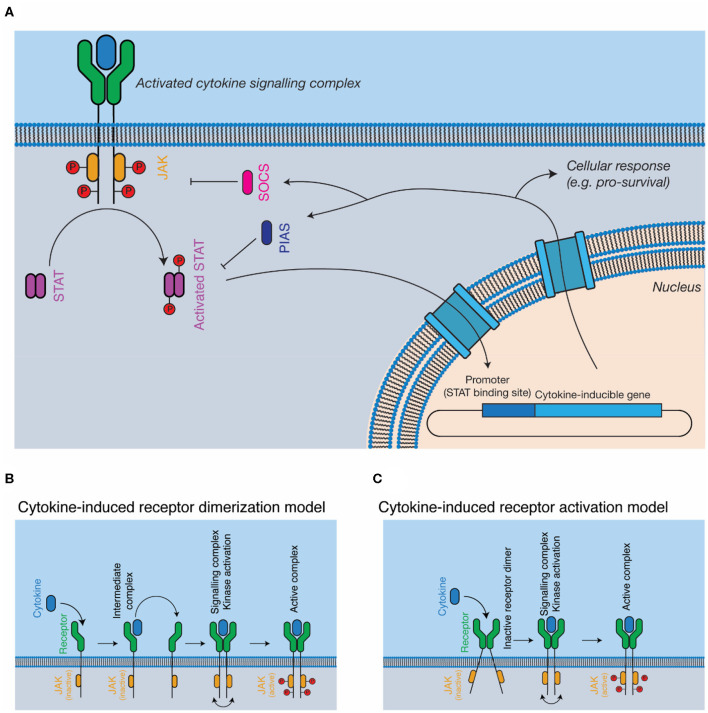
Cytokine signal transduction. **(A)** General schematic of the JAK-STAT pathway. Cytokine binding results in the activation of intracellular kinases (JAKs) that phosphorylate and activate STATs, which subsequently translocate to the nucleus, resulting in altered gene expression, and negative feedback on the pathway through the SOCS proteins. **(B,C)** Models for complex activation. Cytokines are thought to either, **(B)** dimerise receptors on the cell surface, resulting in kinase autophosphorylation and activation or **(C)** bind to pre-dimerised receptors on the cell surface, resulting in receptor activation through conformational alterations of the receptor dimer.

The JAK-STAT pathway is the most well-studied pathway activated in response to cytokines (Figure 2A). The major components of the pathway are cytokine, cytokine receptor, kinase (i.e., JAK), signal transducer (i.e., STAT), and negative feedback regulators (i.e., SOCS). JAKs are associated with the cytoplasmic domains of signal-transducing cytokine receptors and consist of four domains, a kinase domain, pseudokinase domain, 4.1 ezrin radixin moesin (FERM) domain, and Src homology 2 (SH2) phosphotyrosine-binding domain. The pseudokinase domain regulates the kinase domain (77), with the term “Janus kinase” referring to the presence of two kinase domains, real and pseudo, named for the two-faced Roman god (21). The FERM/SH2 domains form a single structural unit (78, 79), and are responsible for interacting with the cytokine receptor, through defined motifs on the receptor, termed Box 1 and Box 2 (80). Cytokine binding results in the activation and phosphorylation of the kinases, which then phosphorylate the cytokine receptor at STAT binding sites, serving to recruit STATs. Bound STATs are themselves phosphorylated, resulting in the activation of the STAT dimer, its translocation to the nucleus, and the expression of cytokine responsive genes. Importantly, different kinases are associated with different cytokine receptors—for example, the IFNα/β receptor primarily uses tyrosine kinase 2 (TYK2) (22) and β_c_ primarily uses JAK2 (81). Furthermore, different receptor-kinase complexes result in activation of different STAT proteins—for example, STAT1/2 for IFNα/βR (22), STAT5 for β_c_ (81), leading to different gene expression programs in response to signaling.

The SOCS proteins, which are expressed as a consequence of cytokine activation, negatively regulate the pathway (27). The SOCS proteins recruit the E3 ligase, Cullin5, resulting in the degradation of the receptor complex in the proteasome (82, 83). Two SOCS proteins, SOCS1 (84) and SOCS3 (85), also directly inhibit the kinase activity of the JAKs. The protein inhibitor of activated STAT (PIAS) proteins inhibit the activity of STAT through mechanisms that include directly blocking STAT interaction with nuclear DNA (86, 87). Several phosphatases act as negative regulators of signaling, such as the SH2-domain containing phosphatases, SHP1 and SHP2 (88, 89) and protein-tyrosine phosphatase (PTP) 1B (90). The lymphocyte adaptor protein, Lnk, serves as an additional negative regulator of signaling by several cytokines that signal using JAK2 (91).

The exact mechanisms by which cytokine engagement triggers signal transduction remain unclear and are the subject of active investigation. In the classical model of cytokine signaling, dimerization of signal transducing receptors simply brings the associated JAKs close enough in proximity to phosphorylate each other in trans (44, 92) (Figure 2B). However, several cytokine receptors, including GHR (43, 93), EPOR (94), and gp130 (95, 96) have been shown to exist as preformed dimers at the cell membrane (Figure 2C). Investigations of GHR suggest that cytokine binding results in a rearrangement of the transmembrane α-helices of the receptor, a conformational change that lifts pseudokinase domain mediated inhibition of the JAKs (43, 93). Determining the universality of such a mechanism will require the study of additional cytokine receptors, particularly those that signal through more complex hetero-dimeric or larger signaling complexes.

In addition to the JAK-STAT pathway, cytokines can utilize alternative signaling pathways, including the mitogen-activated protein kinase (MAPK) pathway, and the phosphoinositide 3-kinase (PI3K) pathway (81). The multi-adaptor protein SH2 domain containing tyrosine phosphatase (SHP2) interacts with several cytokine receptors and provides the link between the receptors and the MAPK pathway (97). Signaling through these pathways is generally less well understood than the JAK-STAT pathway.

## The IL-6 Family of Cytokines

**Figure 3:**
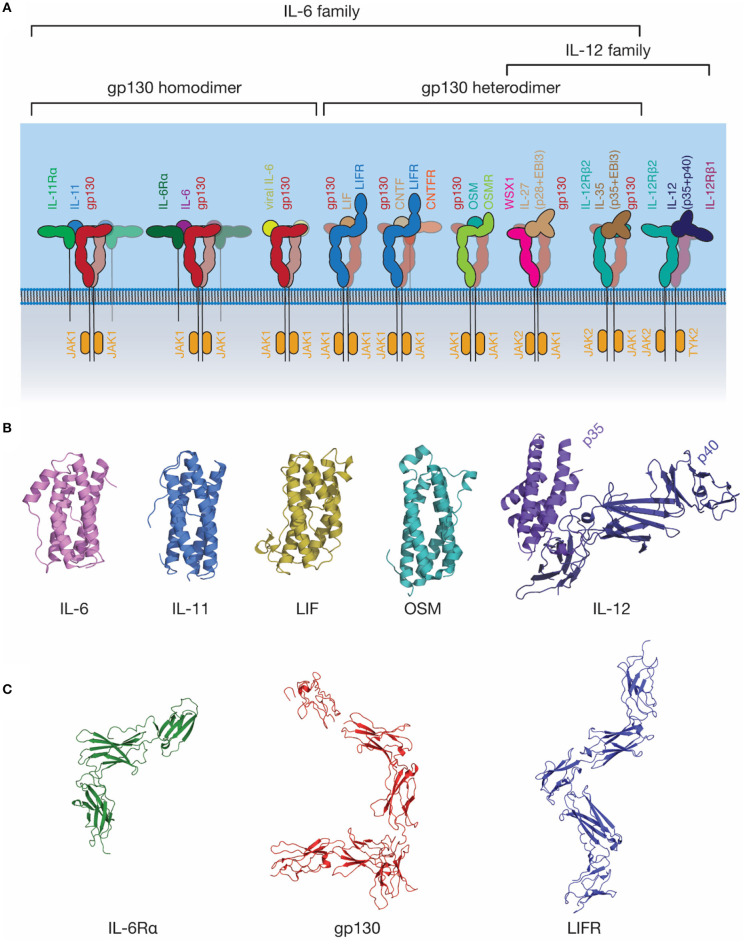
The IL-6 family of cytokines. **(A)** A schematic representation of selected IL-6 and IL-12 family cytokine-receptor complexes, illustrating the diversity in the stoichiometry of signaling complexes employed. Indicative JAK family members utilized by each signal transducing receptor are shown. **(B)** The structures of several IL-6 and IL-12 family cytokines: IL-6 [PDB ID: 1ALU (45)]; IL-11, [PDB ID: 4MHL (98)]; LIF [PDB ID: 1LKI (99)]; OSM [PDB ID: 1EVS (100)]; IL-12 [PDB ID: 1F45 (54)]. **(C)** The structures of extracellular domains of IL-6 family cytokine receptors: IL-6Rα [PDB ID: 1N26 (101)], the common signal transducing receptor, gp130 [PDB ID: 3L5H (58)] and the receptor for LIF and several other IL-6 family cytokines, LIFR [PDB ID: 3E0G (65)].

The IL-6 family of cytokines is one of the largest cytokine families (Figure 3). These cytokines are unified by the near-universal use of the shared signal transducing receptor, gp130. The exception is IL-31, which uses the related receptor IL-31Rα, also known as gp130-like receptor (GPL) (102, 103). The distinct biological activity of IL-6 family cytokines is controlled by the restricted expression of the cytokine-specific receptors, such as IL-6Rα and IL-11Rα by a limited subset of cell types (104). Several cytokines can bind IL-6Rα in addition to IL-6, including CNTF (105), the IL-27 subunit IL-27p28 (also known as IL-30) (106), a IL-27p28 fusion with cytokine-like factor (107), and human herpes virus 8 IL-6 (vIL-6) (108), a viral analog of IL-6 with ~25% sequence identity to mammalian IL-6 (109). Receptor promiscuity is thus a common feature of the IL-6 family.

### The Structure of IL-6 and Its Receptors

**Figure 4:**
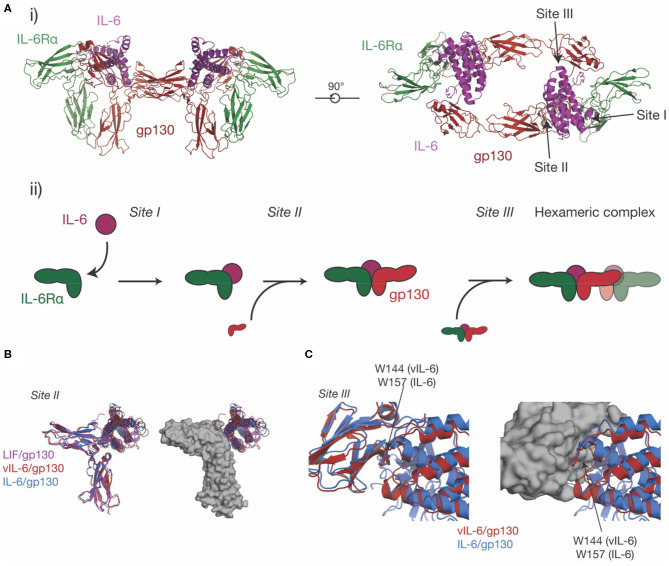
The structure of the IL-6 signaling complex. **(A)** (i) Two views of the structure of the hexameric IL-6/ IL-6Rα/gp130 complex [PDB ID: 1P9M (68)]. The three binding sites on the cytokine (site-I, binding IL-6Rα, site-II, binding gp130, site-III binding gp130), are indicated in the figure. The stepwise assembly of the complex is shown in (ii), with the interactions mediated by each of the binding sites indicated. **(B)** The binding of three IL-6 family cytokines to the CHR of gp130, IL-6, vIL-6 [PDB ID: 1I1R (67)] and LIF [PDB ID: 1PVH (76)]. The three cytokines do not induce significant rearrangements in the CHR of gp130 but adopt a different pose on the CHR and bind different regions in the surface of gp130. **(C)** The binding of IL-6 and vIL-6 to gp130 D1, the interaction that forms the hexameric complex. The two cytokines engage gp130 D1 in an analogous way. The key tryptophan “hot-spot” residue in site-III is indicated.

IL-6 was initially identified under several names in the 1980s (110, 111) as a protein involved in B-cell differentiation (112), a plasmacytoma growth factor (113), and a protein involved in the induction of acute phase proteins in the liver (114). Subsequent cloning of these proteins showed that they were all identical, thus they were given a common name, IL-6. IL-6 is the most well-characterized member of this family structurally, with crystal structures of IL-6 solved in 1997 (45, 115), the structure of IL-6Rα solved in 2002 (101), and the structure of the IL-6 signaling complex solved in 2003 (68) (Figures 3B,C, 4A). IL-6 is a typical four-α helical bundle cytokine, with the expected up-up-down-down arrangement of α-helices, with an additional, short α-helix in the CD loop (Figure 3B). The extracellular region of IL-6Rα consists of three domains (101), an N-terminal Ig-like domain, and two Fn3 domains, which form the IL-6 binding CHR (Figure 3C). The N-terminal Ig domain adopts a distorted Ig-like fold, and is dispensable for cytokine binding and biological activity (60, 68), although there is some evidence that it is required for correct trafficking of the receptor (60). IL-6 binds the surface formed by the two Fn3 domains, D2 and D3, comprising the CHR (68). C-terminal of the structured extracellular domains (D1-D3), there is a long linker region (52 residues), predicted to be disordered, that appears to function as a spacer in the signaling complex between the structured extracellular domains and the membrane (116–118).

Gp130 is the common signal transducing molecule for nearly all IL-6 family cytokines, and some cytokines in the closely related IL-12 family. It was first identified in 1989 (119) as the component of the IL-6 signaling complex involved in signal transduction, and subsequently cloned in 1990 (120). Following this, gp130 was recognized as being a common component of the IL-11 (121), OSM (122), LIF and CNTF (123) signaling complexes. Structures of the CHR domains of gp130 became available in 1998 (124), and the full extracellular region of gp130 in 2010 (58) (Figure 3C). The extracellular domains of gp130 are those of a typical “tall” cytokine receptor, consisting of six domains, an N-terminal Ig-like domain, and five Fn3 domains (58). The first three, membrane-distal domains (D1-D3) are involved in cytokine recognition and complex formation, and are sufficient to bind cytokines and form a complex in solution (68, 76). The membrane-distal domains are also directly involved in gp130 activation, with oncogenic mutations that result in cytokine-independent activation of gp130 clustering in D2 (125). These mutations are thought to act by disrupting the D2/D3 interdomain linker, allowing the receptor to adopt an active conformation in the absence of ligand (126).

The three membrane proximal domains of gp130 (D4-D6) are not directly involved in binding the cytokine, but are required for signal transduction, as deletion of any of the domains results in an inactive receptor (127). Electron microscopy shows that the membrane-proximal domains are involved in the correct orientation of the intracellular kinases for signal transduction (65, 70, 71, 128). In addition to the extracellular domains, gp130 contains a large intracellular domain, which is involved in binding molecules required for signal transduction. Structurally, little is known about the intracellular domain of gp130, although NMR studies have shown that the isolated intracellular domain is disordered (65). JAK1, which mediates intracellular signaling, has been shown to bind gp130 at the Box 1 motif in the intracellular domain of gp130 (80). STAT3 (17, 129) and STAT1 (130) bind at C-terminal phosphotyrosine residues in the intracellular domain of gp130 (131). Specifically STAT3 utilizes Tyr767, Tyr814, Tyr905, and Tyr915, while STAT1 utilizes Tyr905 and Tyr915 (132). SHP2 is also recruited by gp130 at the intracellular domain (133), interacting with Tyr759 providing the link between gp130 and the MAPK pathway (134). The same Tyr759 allows for SOCS3 regulation of cytokine signaling (27, 85).

### The Structure of the IL-6 Signaling Complex

Prior to the determination of the structure of IL-6 in complex with the cytokine binding domains of its receptors (Figure 4Ai) (68), there was extensive evidence from analytical ultracentrifugation and electrophoresis that the complex was hexameric, comprising two copies each of IL-6, IL-6Rα, and gp130 (135–137). Concurrently, mutagenic studies identified three binding sites on IL-6 (136), which were later confirmed in the structure of the complex (68). Site-I is responsible for binding IL-6Rα, site-II is responsible for binding the first molecule of gp130, and site-III is responsible for binding the second molecule of gp130, resulting in the formation of the hexameric complex (Figures 4Ai, ii). Site-I and site-II are positioned on the cytokine in a broadly analogous manner to GH and form a similar trimeric complex, with IL-6 binding the CHRs of IL-6Rα and gp130 (33) (Figure 4Ai). The distinct cytokine:Ig domain interaction between the cytokine and D1 of gp130 is unique to IL-6 family cytokines (138). This interaction is formed by site-III on the cytokine. The complex is formed by ten interdependent interfaces between IL-6 and the two receptors, and between the receptors, with the earlier binding events creating composite binding surfaces to enable subsequent receptor recruitment. The structure of the IL-6 signaling complex has aided drug design studies (139, 140), showing its value in the design of novel therapeutics.

The site-II/CHR region of gp130 is involved in the binding of all gp130-binding cytokines. Alongside the structure of the IL-6 signaling complex, structures were solved of vIL-6 in complex with gp130 (67) and LIF in complex with gp130 (76). All three cytokines engage the CHR of gp130 *via* the site-II region of the cytokine (Figure 4B). The structures showed that vIL-6, IL-6, and LIF engage different but overlapping binding regions in the CHR of gp130, with the three cytokines adopting different poses. A key residue in site-II of gp130, Phe169, forms important interactions with IL-6, vIL-6, and LIF. Surprisingly, the cytokine binding surface of gp130 is relatively rigid, and does not significantly change conformation in response to the binding of different cytokines (76). The CHR of gp130 presents a large, chemically diverse binding surface and the different regions engaged by IL-6, vIL-6, and LIF result in each cytokine/gp130 interaction displaying different thermodynamic properties (76). The size and “thermodynamic plasticity” (76) of the CHR of gp130 is thought to result in its promiscuous binding to multiple cytokines (33, 76).

IL-6 and vIL-6 interact with the Ig-like domain D1 of gp130 through site-III on the cytokine. The interactions between IL-6/gp130 D1 and vIL-6/gp130 D1 are broadly analogous (Figure 4C). In both complexes, a conserved tryptophan is the key hydrophobic “hot spot” residue (Trp157 in human IL-6, Trp144 in vIL-6), providing ~25% of the buried surface area at site-III. Likewise, the N-terminus of gp130 forms a short mainchain-mainchain interaction with the AB loop of the cytokine (67, 68). The site-III interface on gp130 D1 is otherwise relatively chemically and structurally featureless (33), providing a low-affinity binding surface that is reliant on prior interactions with other receptors for stable complex assembly. An interaction similar to the gp130-D1 interaction is formed by LIF with the Ig-D3 and Fn3-D4 of LIFR, although this interaction buries more surface area and forms more polar interactions (59).

No structural data are available for the gp130 binding epitopes of any IL-6 family cytokines other than vIL-6, IL-6, and LIF. Mutagenesis of gp130 shows that IL-11 and IL-6 both require D1 of gp130 for signaling, and bind a similar epitope in the CHR (141). Monoclonal antibodies against gp130 have been developed that antagonize signaling through specific cytokines, including IL-11 and IL-6-specific neutralizing antibodies, suggesting that each cytokine engages gp130 using a structurally different mechanism (142); however, the structural basis of this specificity is currently unknown.

No high-resolution structures are available of the complete extracellular regions of any IL-6 family cytokine complex. All complexes described above comprise heavily truncated forms of the receptors to facilitate crystallization. Electron microscopy (both cryogenic and negative stain) has been used to study several complexes, including the IL-6 complex (70, 71), the LIF complex (65), and the IL-11 complex (128). The resolution in these studies is insufficient to resolve structural detail of the complex, although they reveal a common “doughnut-shaped” architecture, with the “legs” of the tall cytokine receptors, LIFR and gp130, bent to create a complex with a hole in the middle. The details of any contacts between the membrane proximal domains of the receptors in these complexes remain to be elucidated and will require the determination of high-resolution structures of the complete extracellular regions of the complexes.

### Alternative Mechanisms of IL-6 Family Signaling

**Figure 5:**
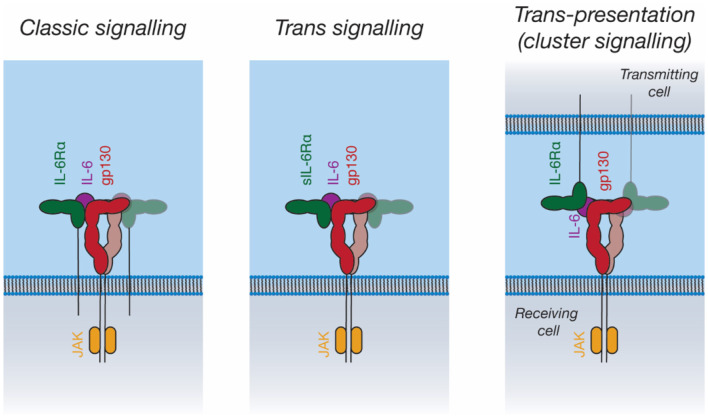
Signal transduction by IL-6. IL-6 can activate intracellular pathways in three ways: “Classic” signaling, in which IL-6 binds to membrane-bound IL-6Rα, and subsequently binds to membrane-bound gp130 on the same cell; “trans-signaling”, in which IL-6 binds to soluble IL-6Rα, subsequently binding membrane-bound gp130; and “trans-presentation,” in which IL-6 binds membrane-bound IL-6Rα on a “transmitting cell” and subsequently engages gp130 on a neighboring (“receiving”) cell, activating intracellular signaling pathways in the receiving cell.

In addition to “classic” IL-6 signaling through membrane-bound IL-6Rα and gp130, IL-6 can also bind a soluble form of IL-6Rα (sIL-6Rα). The IL-6/sIL-6Rα complex can then engage membrane-bound gp130, allowing the stimulation of cells that do not express IL-6Rα, a process known as *trans*-signaling (119, 143) (Figure 5). IL-6 *trans*-signaling is implicated in IL-6 mediated inflammation (143). sIL-6Rα is generated through alternative splicing (144) and through cleavage of the intact receptor by the membrane-bound metalloproteases, ADAM10 and ADAM17, resulting in shedding of the extracellular receptor domains (143). The physiological antagonist of *trans*-signaling is soluble gp130 (sgp130), which can bind to the sIL-6Rα/IL-6 complex extracellularly, thereby neutralizing its cellular activity (145).

IL-11 *trans*-signaling has recently been identified (146). The membrane metalloprotease ADAM10 can cleave IL-11Rα to produce sIL-11Rα, which can engage IL-11 and gp130 in an analogous manner to IL-6/sIL-6Rα (146). To date, no clear biological role has been ascribed to IL-11 *trans*-signaling. In diseases shown to be driven by classic IL-11 signaling, for example gastrointestinal cancers, it has been shown that there is no role for IL-11 *trans*-signaling (147). Likewise, the loss of classic IL-11 signaling is associated with defects in embryo implantation; however, the inhibition of IL-11 *trans*-signaling in mice does not result in infertility (148). Fusion proteins of IL-6 with IL-6Rα and IL-11 with IL-11Rα (“hyper-IL-6 and hyper-IL-11”) are used to mimic *trans*-signaling experimentally (149, 150).

Recent studies have proposed a third IL-6 signaling mechanism, *trans*-presentation, whereby IL-6 binds IL-6Rα on a “transmitting cell,” which then presents the IL-6/IL-6Rα complex to gp130-expressing cells (Figure 5) (151, 152). This was shown to be critical for the differentiation of T_H_17 T helper cells, where IL-6/IL-6Rα is presented in *trans* by dendritic cells (151). *Trans*-presentation has also been shown to be possible for IL-11Rα, however a defined biological role for this has not been identified (152). *Trans*-presentation of IL-6 family cytokines has not yet been characterized structurally; such a signaling mode would require large rearrangements of the IL-6 signaling complex components. Other cytokines such as IL-2 (153) and IL-15 (154) can utilize similar *trans*-presentation mechanisms, where dendritic cells present the cytokine in *trans* to antigen-specific T-cells (48, 155).

### Related Cytokine Families

#### The IL-12 Family of Cytokines

The IL-12 family of cytokines is closely related structurally to the IL-6 family of cytokines, indeed, it has been suggested that a clear distinction between the two families is almost impossible to define (156). In contrast to the majority of the IL-6 family, all IL-12 family cytokines consist of two subunits, a smaller four-α helical subunit, and a larger all-β protein cytokine receptor subunit, which is analogous to the α-receptors for IL-6 and IL-11. For example, IL-12 consists of two subunits, p35, analogous to a four-α helical bundle cytokine, and p40, which resembles a class I cytokine receptor (Figure 3B) (54).

IL-27 and IL-35 are two IL-12 family cytokines that utilize gp130 as a signal transducing molecule and, thus, are also grouped as members of the IL-6 family (Figure 3A) (157, 158). IL-27 consists of a complex of IL-27p28 and Epstein–Barr virus-induced gene 3 (EBI3) that signals through a heterodimer of WSX1 and gp130 (Figure 3A) (157). In addition to this complex, IL-27p28 may utilize IL-6Rα as the cytokine-receptor subunit to signal through a gp130 dimer (106). IL-27p28 was also shown to antagonize IL-6 and IL-27 signaling through gp130, but not OSM signaling, suggesting that IL-27p28 may compete with cytokines that bind D1 of gp130 (159). IL-35 can signal using a heterodimer of IL-12Rβ2 and gp130, or homodimers of either IL-12Rβ2 or gp130; however, the molecular mechanisms underpinning this promiscuity are currently unclear (158). Broadly, these findings suggest an evolutionary relationship between the IL-6 and IL-12 families of cytokines and underscore the promiscuity of cytokine receptors in the IL-6/IL-12 superfamily.

#### Domeless

A distant homolog of gp130 has been identified in *Drosophila melanogaster*, the receptor *domeless* (*dome*) (160), which is the likely evolutionary ancestor to all IL-6 family cytokine receptors (161). *Dome* shares a similar domain structure to gp130 and LIFR, and has a putative CHR, albeit with low sequence identity to the CHR of gp130. A putative ligand for *Dome, Unpaired-3* (*Upd3*) (162) has also been identified, alongside JAK kinases (*Hopscotch*) and STAT transcription factors (*Marelle*) (163). The *Dome-Hopscotch* pathway has been shown to have several roles in *Drosophila* physiology, including in responding to bacterial infection (164), in oogenesis (164), in hemocyte proliferation (165), and in tissue development (166, 167), showing that cytokine pleiotropy is a common feature in metazoans. Neither *dome* or *Upd3* have been studied structurally, although recombinant *Upd3* has been produced, and has been shown by circular dichroism spectroscopy to have a predominately α-helical secondary structure (168). Zebrafish possess a mammalian-like cohort of cytokines, with relatives of all extant mammalian cytokine families present, suggesting that an increase in diversity of cytokines and receptors occurred with the evolution of the adaptive immune system in vertebrates (169, 170).

## Biological Roles of IL-11

IL-11 was first identified in 1990, following the discovery of a protein factor that stimulated a murine plasmacytoma cell line previously thought to be IL-6 dependent (171). The following year, IL-11 was also identified as a factor secreted from a bone marrow derived cell line culture, which inhibited adipogenesis in preadipocytes (172, 173), thus the pleiotropic nature of IL-11 signaling was appreciated early. While there was a flurry of activity surrounding IL-11 in the 1990s, there has been less research activity since. However, in the last decade there has been a renewed interest in IL-11 following its emerging role in numerous diseases.

### Structure of IL-11 and Its Receptors

**Figure 6:**
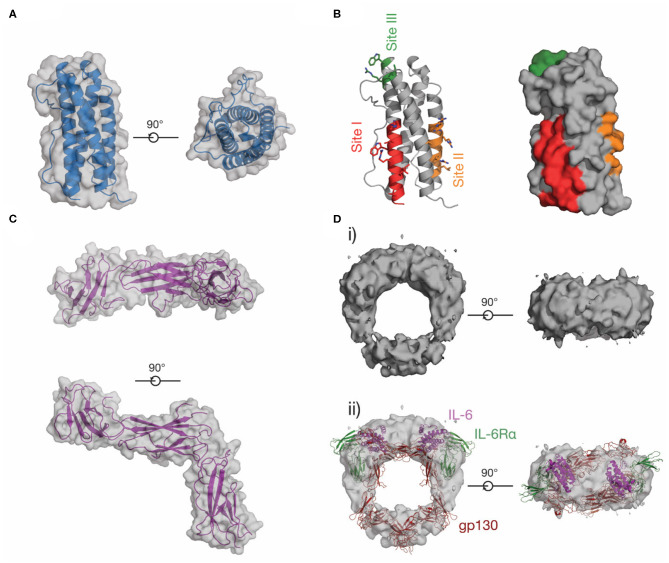
The structure of IL-11. **(A)** Two views of the structure of IL-11 [PDB ID: 6O4O (174)]. **(B)** Receptor-binding sites on IL-11, which have been identified through mutagenic studies on human and mouse IL-11. **(C)** Two views of the structure of the extracellular domains of IL-11Rα [PDB ID: 6O4P (174)]. **(D)** A low-resolution EM map of the extracellular IL-11 signaling complex [EMD-1223 (128)] (i), additionally shown overlaid with a model of the extracellular IL-6 signaling complex, (ii) [generated from PDB IDs: 3L5H (58), 1P9M (68)].

In contrast to IL-6, LIF and other IL-6 family cytokines, little was previously known about the structure of IL-11 or IL-11Rα. We reported the first crystal structure of IL-11 in 2014 (98) and have recently reported a higher-resolution structure of the cytokine (Figure 6A) (174). Our structures show that IL-11 is ~5 Å longer than IL-6, suggesting differences in binding mode and geometry within the signaling complex. Likewise, the IL-11Rα binding site (site-I) and the first gp130 binding site (site-II) of IL-11, previously identified through mutagenesis (175, 176), are different in chemical character to IL-6, with site-I more hydrophobic (Figure 6B). Our recent structure of IL-11Rα (Figure 6C) (174) revealed that the cytokine binding site of the receptor is more hydrophobic in character than IL-6Rα, consistent with the corresponding site of IL-11 and suggesting distinct mechanisms of cytokine engagement.

No high-resolution structural data for the IL-11 signaling complex are currently available in the literature, although sequence analysis (121, 177) and our structural data (174) show that IL-11Rα and IL-6Rα are structurally similar. The IL-11 signaling complex, like the IL-6 signaling complex, is thought to be hexameric, as shown by immunoprecipitation and electrophoresis (178). Contemporaneous mutagenic studies (175, 176, 179) also identified site-I, II, and III on IL-11 (Figure 6B), suggesting that IL-6 and IL-11 form an active signaling complex using a broadly similar mechanism. A low-resolution (~30 Å) cryoEM density map of the IL-11 signaling complex extracellular domains (128) (Figure 6D) shows that the overall arrangement of the complex is broadly similar to the IL-6 signaling complex (Figure 6Dii), although the details of complex formation were not clear at this resolution. We have recently solved structures of the IL-11 signaling complex that provide high resolution detail of the assembled complex (unpublished).

### IL-11 in Hematopoiesis

Early studies of IL-11 revealed that it was a potent hematopoietic factor, acting synergistically in culture with other cytokines, such as IL-3 (180, 181) and IL-4 (182). In particular, IL-11 was found to have a role in megakaryocytopoiesis, causing the maturation of megakaryocytes, large cells which form platelets (181). In mice, IL-11 alone is a potent hematopoietic stimulator following radiation therapy and chemotherapy, and markedly increases platelet counts (183). Recombinant IL-11 is approved by the FDA to treat thrombocytopenia following radiation treatment in humans (184), and is commonly prescribed to breast cancer patients. In addition to its well-characterized role in megakaryocytopoiesis, IL-11 has other roles in hematopoiesis (185), for example, in lymphopoiesis (186), in erythropoiesis (187), and in myelopoiesis (188).

### IL-11 in Bone Development

IL-11 signaling has been shown to promote osteoblast differentiation, and thus bone formation, with IL-11Rα knockout mice showing abnormal craniofacial features (189–191). In humans, mutations in the genes for IL-11 and IL-11Rα are associated with a reduction in height (192, 193), suggesting that IL-11 signaling has a role in regulating growth. Likewise, a genetic variant in the gene for IL-11, resulting in a substitution mutation (R112H), is associated with osteoarthritis and a reduction in height (192, 194). Biochemical characterization of the mutant cytokine has shown that it does not alter the biological activity of IL-11, but compromises the stability of the protein (195).

Over the past decade, a number of studies have identified mutations in the gene for IL-11Rα, which result in a genetic disease associated with craniosynostosis (196–198). Craniosynostosis is a condition in which bone plates in the skull fuse too early, resulting in facial abnormalities and an abnormally shaped skull. The disease is rare, and has been found in families with diverse geographic origins (196). Generally, the disease occurs as a result of point substitution mutations in the extracellular domains of IL-11Rα (196, 199), and many of these mutations are situated in regions distant from the putative cytokine or receptor binding sites. Several of the mutations have been shown to impair correct processing and surface expression of the receptor (199). Molecular dynamics simulations using our IL-11Rα structure indicate that some mutations destabilize the receptor and may have indirect effects on the cytokine binding region (174).

### IL-11 in The Lung

IL-11 is highly expressed as a consequence of viral induced asthma (200), and overexpression of IL-11 in the airways of mice results in remodeling of the airways, inflammation and asthma-like symptoms (201). Subsequent studies have shown that IL-11 signaling is critical for a T_H_2-mediated inflammatory response in the lung (202), and that inhibition of IL-11 signaling in the lung alleviates inflammation, implying that IL-11 signaling is a therapeutic target in asthma (203). Similarly, IL-11 has been shown to drive lung inflammation in a murine model of *Mycobacterium tuberculosis* infection (204).

### IL-11 in Reproduction

Female knock-out mice lacking the gene for IL-11Rα are infertile, and cannot undergo the uterine transformations required for embryo survival (205). Likewise, IL-11 and IL-11Rα have been localized to reproductive tissues during early pregnancy in primates, suggesting a role in placentation and decidualization (206). Related to this, inhibition of IL-11 signaling impairs decidualization and prevents pregnancy in mice, suggesting that therapeutic inhibition of IL-11 may be a potent contraceptive (207). Defects in the production of IL-11 have also been associated with anembryonic pregnancy, a cause of miscarriage (208). IL-11 signaling inhibits and regulates invasion of extravillous trophoblasts, cells which are key in placentation for the formation of blood vessels (209–211). Thus, elevated IL-11 is associated with preeclampsia, a disease where placentation is impaired, resulting in hypertension (211). Together, these studies suggest that IL-11 has key roles in driving the tissue transformations that occur as a result of pregnancy.

### IL-11 in Fibrosis

IL-11 has been implicated in fibrosis of the heart (212), liver (213), and lung (214, 215). Fibrosis is the generation of excess connective tissue, and is a hallmark of several diseases, including late-stage cardiovascular disease, and liver diseases such as non-alcoholic liver disease. In the heart, IL-11 has recently been identified as a key fibrotic factor, acting downstream of the main fibrotic factor TGFβ1, driving fibrotic protein synthesis in an autocrine manner (212). IL-11 has a similar role in driving inflammation and fibrosis of the liver (213). Interestingly, in both cases, the effect has been shown to be driven by non-canonical signaling via the MAPK/ERK pathway, rather than *via* the JAK-STAT pathway. Surprisingly, canonical IL-11 signaling via STAT3 has previously been ascribed a cardioprotective role, inhibiting cardiovascular fibrosis and preventing cardiovascular remodeling following myocardial infarction (216). These contradictory observations may be a consequence of the source of IL-11 used in either study, as it was shown that human IL-11, previously used to show that IL-11 is cardioprotective, does not activate mouse cardiac fibroblasts, while murine IL-11 strongly activates murine cardiac fibroblasts (212). Alternatively, it may suggest different roles for IL-11 in response to different cardiovascular stresses. More broadly, this may reflect an inadequate understanding of the species-specific effects of IL-11, or differences in signaling in humans as compared to mice.

### IL-11 in Cancer

IL-11 signaling drives several cancer hallmarks (217, 218) including cell survival, metastasis, and invasion (219–221). IL-11 levels are significantly higher in a murine model of gastric cancer (222), and IL-11 is the major factor that drives STAT3 activation and corresponding inflammation in murine gastric and colon cancer models, as well as human cell line xenograph models of these cancers (221). A role for IL-11 signaling in breast cancer has been less well-described, but elevated levels of IL-11 and IL-11Rα are associated with poor patient outcomes (223, 224) and both IL-11 and IL-6 are associated with breast cancer metastasis into bone (225). IL-11 is also associated with endometrial cancer, and is associated with increasing tumor grade (226). Elevated levels of IL-11 are found in several other types of cancer, including pancreatic cancer (227), skin cancer (228), and bone cancer (229), although a precise role for IL-11 signaling in many of these cancers remains to be defined.

## Therapeutic Targeting of IL-6 Family Cytokine Signaling

Given the role of cytokine signaling in numerous pathological conditions there is broad interest in the development of therapeutic agents that block their activity. Generally, inhibition can occur at several points in the cytokine signaling pathway—either by preventing the protein-protein interactions on the cell surface, or by targeting components of the signal transduction machinery within the cell. Conversely, recombinant cytokines can also be used to therapeutically boost cytokine signaling. Here we provide an overview of several approaches to therapeutically modulate cytokine signaling that are in development, as well as those currently used in the clinic. We focus our discussion on how advances in these areas may inform the design of IL-11 signaling inhibitors suitable for clinical use.

### Small Molecules

#### Inhibitors of Intracellular Signal Transducing Proteins

**Figure 7:**
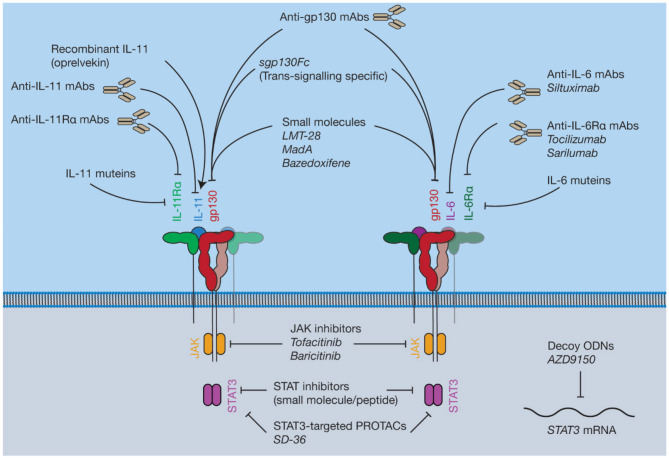
Pharmacological approaches to target IL-6 and IL-11 signaling. Current inhibitors of IL-6 and IL-11 signaling include protein antagonists such as cytokine mutants and antibodies, small molecule protein-protein interaction (PPI) inhibitors targeting gp130, recombinant IL-11, small molecule inhibitors of proteins in the intracellular JAK-STAT pathway, and decoy oligodeoxynucleotides (ODNs) targeting the *STAT3* mRNA.

JAK inhibitors are widely used, orally bioavailable, small molecules for the treatment of blood cancers and inflammatory diseases (230) (Figure 7). Six JAK inhibitors are used clinically, with several in development. For example, the JAK1/2 selective inhibitor ruxolitinib (231) is used to treat a group of rare blood cancers associated with an activating mutation in JAK2. Similarly, tofacitinib (non-selective) and baricitinib (selective for JAK1/2) are JAK inhibitors used to treat the inflammatory disease rheumatoid arthritis (232, 233). JAK inhibitors are now undergoing clinical trials for a broader array of inflammatory diseases (234). Challenges with developing JAK inhibitors are largely a consequence of the inherently non-specific nature of the drugs. Moreover, JAK inhibition may be associated with severe side effects, including opportunistic viral infections, likely a consequence of inhibition of interferon-mediated protective antiviral signaling (235). Similarly, due to the central roles of cytokine driven JAK activation in hematopoiesis, JAK inhibitors have been noted to cause mild anemia and neutropenia (236, 237). Despite this, JAK inhibitors are widely used, and efforts to develop novel JAK inhibitors, particularly inhibitors that are selective for a specific kinase, are ongoing.

Inhibitors of STAT activity are in various stages of development (238). Phase I and II trials have been conducted on several drug candidates targeting STAT3, although the results are pending (239, 240). These inhibitors are generally peptides or small molecules designed to prevent STAT dimerization (241, 242), or decoy oligodeoxynucleotides (ODNs) designed to target expression of the STAT gene directly (243). Recently, a small-molecule proteolysis targeting chimera (PROTAC), SD-36 (244), which selectively targets STAT3 over other STAT family members, has been described. Direct inhibition of activated STATs is at a less advanced stage compared to kinase inhibitors, or drugs targeting the cytokine/receptor interaction directly, with current inhibitors having low potency and poor pharmacokinetic properties (245). For example, curcumin, an extract of the turmeric plant, *Curcuma longa*, has been used in traditional medicine for centuries for its anti-inflammatory properties (246). Mass spectrometric and computational docking studies have shown that curcumin directly interacts with STAT3 to inhibit phospho-STAT3 dimerization (247). Several *in vitro* studies demonstrate that curcumin is an inhibitor of STAT3 signaling (247, 248). However, the use of curcumin as a drug candidate or treatment is controversial (246, 249). Generally, direct targeting of STATs may not have clear benefits over existing therapeutic strategies, such as JAK inhibitors, which may hinder clinical uptake of STAT inhibitors.

#### Inhibitors of Signaling Through gp130

Several small molecules have been described that are believed to bind to gp130 and inhibit the protein-protein interactions (PPIs) that result in complex formation (Figure 7). Despite the challenges of targeting PPIs, as they present large flat binding surfaces (250), small molecule modulation of PPIs is potentially invaluable therapeutically. Small molecule inhibitors could be more specific for the inhibition of signaling through individual cytokines compared to JAK inhibitors, which modulate the signaling of numerous cytokines. Moreover, PPI-inhibitors would likely be cheaper, orally bioavailable, and have a shorter half-life compared to biologic therapies, which is beneficial in the event of serious adverse events (251).

Madindoline A (MadA), a natural product isolated from *Streptomyces nitrosporeus* culture, is a small molecule shown to specifically inhibit the activity of IL-6 and IL-11 *in vitro* (252). MadA has subsequently been shown to inhibit the action of IL-6/IL-11, but not LIF, in bone resorption and macrophage differentiation (253). Additional studies have shown that MadA binds specifically to gp130, with a low affinity (254). Chemical synthesis of MadA is difficult (255) and it is produced in low yields by bacterial fermentation, limiting its potential as a drug candidate for large scale production.

The small molecule gp130 inhibitor SC144 was identified serendipitously from efforts to design a human immunodeficiency virus (HIV) integrase inhibitor, which would be a potential anti-HIV drug (256, 257). Several candidate HIV integrase inhibitors were highly cytotoxic (258). A library was built from these cytotoxic molecules (256) and further screening and lead optimization resulted in the synthesis of SC144 (257), which was effective against a variety of cancer models (259). Subsequently, it was shown that SC144 binds gp130 and inhibits the activity of IL-6 and LIF, likely through binding the CHR of gp130, resulting in suppression of cancer growth in human ovarian cancer xenographs (260). Following this initial description of its activity, SC144 has been used by various groups as an experimental tool to block IL-6 signaling through gp130 [see for example (261–263)].

Another small molecule inhibitor that has been shown to bind to gp130, LMT-28, was identified by screening a library of ~1,000 compounds (264). Computational docking suggested that LMT-28 binds to D1 of gp130, and the putative binding region in D1 of gp130 was supported using site-directed mutagenesis (265). Likewise, SPR showed that LMT-28 specifically bound gp130, with a dissociation constant (*K*_D_) of 7.4 μM, and LMT-28 was able to out-compete IL-6/IL-6Rα for gp130 binding (264). LMT-28 has been shown to specifically inhibit IL-6/IL-11 driven cell proliferation, and block IL-6-driven inflammation *in vivo* (264). In contrast, LMT-28 does not inhibit OSM/LIF activity, consistent with a binding site in D1 of gp130 (264).

Bazedoxifene is an FDA-approved selective estrogen receptor modulator used clinically in combination with other drugs to treat osteoporosis in elderly women (266). It was recently shown that bazedoxifene inhibited gp130 signaling, following an *in silico* screen on the IL-6/gp130 site-III interface (139). Bazedoxifene has been shown to suppress STAT3 activation through IL-6, inhibit tumor growth in a murine model of rhabdomyosarcoma, a soft-tissue sarcoma (267), and inhibit the proliferation of IL-6 dependent cell lines (268). Bazedoxifene has also been shown to block STAT3 activation by IL-11 in human cancer cell lines, and reduce the tumor burden in murine models of gastric cancer (140). Bazedoxifene was also shown to inhibit IL-6 signaling in triple negative breast cancer cell lines (269), and in murine models of the inflammatory cardiovascular disease abdominal aortic aneurysm (270). Recently, more efficacious analogs of bazedoxifene have been synthesized (271). Given that bazedoxifene is already used clinically, and thus has an established safety profile, it represents a potential small molecule inhibitor of both IL-11 and IL-6 signaling that could be used therapeutically.

### Biologics

#### Recombinant Cytokines

Generally, with some exceptions, recombinant cytokines have not seen wide use therapeutically. Although rare, long-term treatment with recombinant cytokines can result in the generation of endogenous antibodies against the cytokine (272). More generally, the pleiotropic nature of many cytokines may result in unpredictable and intolerable inflammation-associated side-effects, which could limit the use of recombinant cytokines in the clinic (273, 274).

Recombinant human IL-11 (oprelvekin) was FDA-approved in 1998 (184, 275, 276) for the treatment of thrombocytopenia (low platelet levels) in myelosuppressive chemotherapy, as a substitute for platelet transfusions. Oprelvekin has also undergone a clinical trial for use thrombocytopenia in myelodysplastic syndrome, in which the bone marrow fails to properly mature blood cells (277). Oprelvekin is, however, not widely used, both for reasons of cost (278) and due to toxicity associated with mild anemia, periostitis, edema and in some cases neuropathy (279, 280). This toxicity can be managed by limiting the dose of oprelvekin (281). IL-11 also has anti-inflammatory properties, and oprelvekin has also undergone small clinical trials in inflammatory bowel disease (282) and rheumatoid arthritis (283). Both trials were inconclusive, and no further trials for either of these indications have been published.

#### Monoclonal Antibodies

Numerous monoclonal antibodies (mAbs) are used clinically to target IL-6 signaling (284), for example, the anti-IL-6Rα mAbs tocilizumab (285) and sarilumab (286), and the anti-IL-6 mAb siltuximab (287) are used to treat several diseases including rheumatoid arthritis and kidney cancer (Figure 7). Antibodies targeting IL-6 signaling are generally well-tolerated but have been noted to result in adverse events. For example, long-term clinical trials have noted that tocilizumab treatment can result in opportunistic infection, neutropenia and gastrointestinal disorders (288, 289), likewise infection, fatigue and neutropenia have been noted as potential adverse effects of siltuximab (290). The anti-IL-6 mAb olokizumab is currently undergoing a phase III clinical trial for rheumatoid arthritis (ClinicalTrials.gov identifier NCT02760368). Structures show that the olokizumab Fab blocks site-III of IL-6, preventing formation of the IL-6 hexameric complex (291). Structures have also been solved of two anti-IL-6 Fabs, which bind site-I, mimicking the IL-6/IL-6Rα interaction (292). No structures are available of the FDA-approved anti-IL-6 signaling antibodies in complex with their antigen.

Viral infections, including influenza (293), and severe acute respiratory syndrome (SARS) (294, 295), caused by SARS-coronavirus (CoV), can induce cytokine release syndrome (often referred to as “cytokine storm”), a severe immune reaction frequently associated with elevated serum IL-6 (296, 297). Severe coronavirus disease 2019 (COVID-19), caused by SARS-CoV-2 (298), is associated with elevated serum IL-6 and cytokine release syndrome (299–301). Thus, IL-6 signaling inhibition may be a strategy for managing severe and critical COVID-19 (302). Accordingly, tocilizumab is currently undergoing several expedited clinical trials in severe and critical COVID-19 patients (for example, ChiCTR ID: ChiCTR2000029765, ChiCTR2000030894; ClinicalTrials.gov ID: NCT04315480, NCT04317092, NCT04372186, NCT04320615) (303). Tocilizumab appears to reduce mortality in severe and critical COVID-19 patients (300, 304–307), however in some cases poor outcomes have been noted (308).

Antibodies against IL-11 (214, 309) and IL-11Rα (213, 310, 311) that inhibit IL-11 signaling have been described and patented, although none are clinically available. The mechanisms of action of these antibodies have not been described in the literature.

Antibodies against gp130 have been described (142) that specifically antagonize signaling through a specific cytokine or cytokines, although they are not used in the clinic. The structural basis of this specificity is currently unknown, although epitope mapping studies have been conducted on the antibodies (142, 312), which show that the IL-11-specific mAb, B-P4, binds the membrane proximal region (D4-D6) of gp130 and not at the CHR. The OSM/LIF-specific mAb (B-K5), CNTF-specific mAb (B-P8) and broadly neutralizing mAb (B-R3) bind at the CHR of gp130, presumably sterically interfering with cytokine binding (142, 312).

#### Soluble gp130

Many of the harmful, pro-inflammatory effects of IL-6 signaling are believed to be caused by *trans* IL-6 signaling (143). Soluble gp130 (sgp130) is an antagonist of trans IL-6 signaling (145). Sgp130 fused to an IgG Fc fragment (sgp130Fc, olamkicept) is currently under development as an IL-6 *trans*-signaling specific inhibitor (313). The effect of sgp130Fc treatment has been studied in animal models of a number of inflammatory diseases including several cancers (314, 315), arthritis (316, 317), inflammatory bowel disease (318, 319), and pancreatitis-associated lung inflammation (320). The side effects of existing treatments targeting IL-6 signaling in humans are believed to result from a blockade of classic signaling, resulting in an increased susceptibility to infections, due to the key role of IL-6 signaling in responding to infection (313, 321). In animal models, blockade of IL-6 *trans*-signaling does not alter the IL-6 dependent response to infection (321). Sgp130Fc is currently undergoing phase II clinical trials for colitis (313) (ClinicalTrials.gov ID: NCT03235752; DRKS-ID: DRKS00010101). An anti-*trans*-signaling nanobody has also been developed (322) which specifically recognizes an epitope formed between IL-6 and IL-6Rα, although the structural mechanism behind inhibition has not been described. IL-11 *trans*-signaling has not been ascribed the same biological significance as IL-6 *trans*-signaling, regardless, sgp130Fc is used as a tool to study IL-11 *trans*-signaling (146), and may be a useful therapy in the case that IL-11 *trans*-signaling is found to be pathologically important.

#### Cytokine Mutants and Designer Proteins

In the past decades, systematic mutagenesis or phage display was used to generate antagonistic variants of IL-6, IL-11, and LIF by altering affinity to IL-6Rα, IL-11Rα, LIFR, or gp130 (203, 323, 324). These antagonists generally function by selectively increasing affinity to one cytokine receptor, and decreasing affinity to a second cytokine receptor, allowing the non-signaling competent mutant to compete with endogenous cytokine for its receptor. For example, a LIF mutein (324) was developed using phage display to increase the affinity for LIFR, while incorporating mutations that reduced the affinity for gp130. This enables the LIF mutein to compete with endogenous LIF for LIFR binding, while the LIF mutein has reduced capacity to form signaling complex with gp130, resulting in inhibition of signaling by LIF. A similar approach was used to design an IL-11 mutein (203). The mutein incorporates two sets of mutations, a mutation in site-III to reduce binding to gp130, and mutations in the AB loop intended to increase affinity to IL-11Rα allowing the IL-11 mutein to compete with endogenous IL-11 for IL-11Rα, and reduce signaling through IL-11.

Recently, a novel CNTF signaling agonist, IC7, was designed (325) by substituting site-III on IL-6 with site-III on CNTF (which binds LIFR), resulting in a cytokine that signals through a gp130/LIFR heterodimer, while being dependent on IL-6Rα, a signaling mode which is not used by any known IL-6 family cytokine (325). Recombinant CNTF has undergone clinical trials previously to treat type-2 diabetes (326), however the trials were halted due the potential immunogenicity of recombinant CNTF. IC7 provides a therapeutic benefit in animal models of diet-induced obesity, and was not observed to have any severe inflammatory or immunogenic side-effects, suggesting that IC7 holds promise as a novel cytokine treatment for diabetes (325).

An additional approach to develop cytokine signaling modulators is the use of computationally *de novo* designed proteins. A notable recent example of the use of protein design is in the development of IL-2 signaling modulators (327). *De novo* designed proteins, which have low sequence identity to endogenous cytokines, can reduce the risk of immunogenicity when using recombinant cytokines or cytokine mutants as drugs. The use of *de novo* protein design may allow the development of IL-11 agonists or antagonists with low immunogenicity that are more potent than existing therapies.

## Concluding Statements

As new roles for cytokines in disease are discovered, the development of therapeutics to inhibit their action invariably follows. Our rapidly increasing understanding of the importance of IL-11 signaling in disease underscores its potential as a therapeutic target. However, the development and appropriate characterization of inhibitors of IL-11 signaling has not matured at the same pace. Detailed biophysical and structural information obtained in parallel with pre-clinical testing can greatly facilitate design, specificity, and potency of new cytokine inhibitors, ensuring that the best therapeutics are entered into clinical trials. Thus, improved structural and molecular understanding of the IL-11 signaling complex and current generation inhibitors will be of great benefit for therapeutic development programs targeting IL-11.

## Author Contributions

RM wrote the manuscript and prepared the figures. TP wrote the manuscript. MG wrote the manuscript and supervised the studies. All authors contributed to the article and approved the submitted version.

## Conflict of Interest

The authors declare that the research was conducted in the absence of any commercial or financial relationships that could be construed as a potential conflict of interest.
